# Thioredoxin and Glutaredoxin Systems as Potential Targets for the Development of New Treatments in Friedreich’s Ataxia

**DOI:** 10.3390/antiox9121257

**Published:** 2020-12-10

**Authors:** Marta Seco-Cervera, Pilar González-Cabo, Federico V. Pallardó, Carlos Romá-Mateo, José Luis García-Giménez

**Affiliations:** 1 Centre for Biomedical Research on Rare Diseases (CIBERER), 46010 Valencia, Spain; marta.seco@uv.es (M.S.-C.); Pilar.Gonzalez-Cabo@uv.es (P.G.-C.); federico.v.pallardo@uv.es (F.V.P.); 2 Department of Physiology, Faculty of Medicine and Dentistry, Universitat de València (UV), 46010 Valencia, Spain; 3 Biomedical Research Institute INCLIVA, 46010 Valencia, Spain

**Keywords:** Friedreich’s ataxia, oxidative stress, thioredoxins, glutaredoxins

## Abstract

The thioredoxin family consists of a small group of redox proteins present in all organisms and composed of thioredoxins (TRXs), glutaredoxins (GLRXs) and peroxiredoxins (PRDXs) which are found in the extracellular fluid, the cytoplasm, the mitochondria and in the nucleus with functions that include antioxidation, signaling and transcriptional control, among others. The importance of thioredoxin family proteins in neurodegenerative diseases is gaining relevance because some of these proteins have demonstrated an important role in the central nervous system by mediating neuroprotection against oxidative stress, contributing to mitochondrial function and regulating gene expression. Specifically, in the context of Friedreich’s ataxia (FRDA), thioredoxin family proteins may have a special role in the regulation of Nrf2 expression and function, in Fe-S cluster metabolism, controlling the expression of genes located at the iron-response element (IRE) and probably regulating ferroptosis. Therefore, comprehension of the mechanisms that closely link thioredoxin family proteins with cellular processes affected in FRDA will serve as a cornerstone to design improved therapeutic strategies.

## 1. Introduction

### Thioredoxin and Glutaredoxin Systems in Redox Biology

The thioredoxin family consists of a small group of redox proteins present in all organisms and composed of thioredoxins (TRXs), glutaredoxins (GLRXs) and peroxiredoxins (PRDXs) [1,2]. TRX and GLRX are classically considered general disulfide reductases which catalyze nicotinamide adenine dinucleotide phosphate (NADPH)-dependent reductions of disulfide (S-S) bridges in oxidized proteins [3], and, hence, TRX and GLRX are generally recognized as antioxidant proteins [4]. Additionally, they also catalyze the reduction of PRDXs [5] contributing to the maintenance of cellular redox homeostasis and protein function.

The thioredoxin system contains TRX and thioredoxin reductase (TRXRD) which uses the electrons from NADPH to re-establish the system by reducing oxidized TRX [6]. Advances in the last ten years have demonstrated that TRX can exist in the extracellular compartment, the cytoplasm, the mitochondria and in the nucleus [7]. Both TRX isoforms, the cytosolic and nuclear TRX1 isoform and the mitochondrial TRX2 isoform, are specifically present in each of these subcellular locations, mediating different cellular functions and activating distinct molecular mechanisms including redox balance, cell proliferation and apoptosis, DNA replication, gene expression, and proteasome function, among others [8,9,10]. Originally, human TRX was identified as an extracellular protein performing multiple functions [11], such as chemokine-like activity [12]. Although the precise mechanism that mediates the secretion of TRX still remains unknown, increased levels of extracellular TRXs, for example, in plasma, serum or saliva, have been reported in many pathological conditions associated with oxidative stress and inflammation [13].

In the cytoplasm, the reduced form of TRX1 regulates the redox environment of the cell and also the activity of certain proteins such as apoptosis signal-regulating kinase-1 (ASK-1), which is required for tumor necrosis factor (TNF)-alpha-induced apoptosis [14].

**Figure 1:**
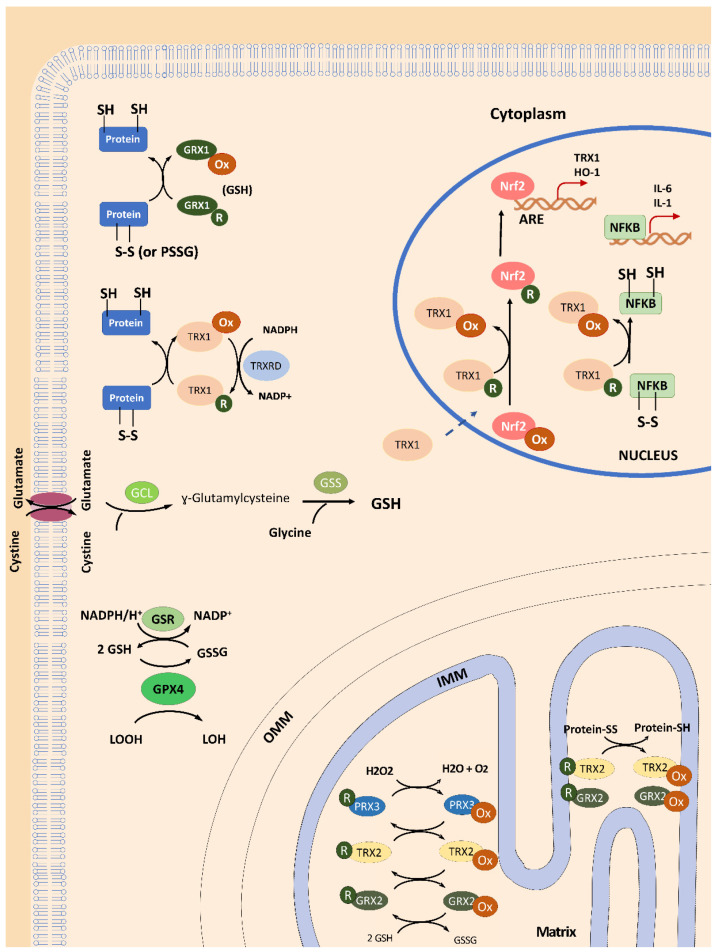
General overview of TRX and GLRX functions in mammalian cells. In the cytosol, glutaredoxins (GLRX1) can reduce protein dithiols through a di-thiol and mono-thiol mechanism or reduce S-glutathionylated proteins (PSSG) in a gluthation (GSH)-dependent process during its activity as disulfide oxireductase. Thioredoxin 1 (TRX1) also acts as dithiol reductase, maintaining the redox homeostasis of the proteins. The reduced form of TRX1 is re-established by thioredoxin reductase 1 (TRXRD1), which uses the electrons from nicotinamide adenine dinucleotide phosphate (NADPH). The NADPH is also important to provide electrons to glutathione reductase (GRX) which can recover the levels of reduced gluthation (GSH) from oxidized form (GSSG), and then be used as cofactor of glutathione peroxidase 4 (GPX4) to reduce lipoperoxides. The reduced form of TRX1 can be translocated to the nucleus and interact with nuclear factor-κB (NF-κB) and activator protein 1 (AP-1) to regulate gene expression. The translocation of the transcription factor NRF2 to the nucleus depends on the cytosolic levels of GSH (which controls the interaction between NRF2 and its modulator Kelch-like ECH-associated protein 1 (Keap1)) and the nuclear TRX1 (which promotes NRF2-DNA binding). Moreover, TRX1-NRF2 interplay facilitates the binding of NRF2 to the antioxidant response element (ARE) to activate the expression of TRX1 and heme oxygenase-1 (HO-1) genes, among others. In addition, TRX1 can regulate the DNA binding activity of NF-κB to its gene targets, such as interleukins IL1 and IL6, and even the *NRF2* gene, thereby also contributing to antioxidant response. In the mitochondria, mitochondrial thioredoxin 2 (TRX2), glutaredoxin 2 (GRX2) and peroxiredoxin 3 (PRDX3) work together to eliminate H2O2, thus maintaining redox control in the mitochondrial matrix environment. Moreover, TRX2 and GRX2 can reduce disulfide bonds in proteins to maintain the redox status of mitochondrial proteins. Glutamate cysteine ligase (GCL); Glutathione synthetase (GSS); Outer mitochondrial membrane (OMM); Inner mitochondrial membrane (IMM).

In the nucleus, TRX1 has been shown to interact with many transcription factors, such as nuclear factor-κB (NF-κB) and activator protein 1 (AP-1), thereby regulating gene expression [9,15,16]. In this regard, Das et al. have described how the expression of the *SOD2* gene, among other antioxidant genes, is induced by TRX [17] through an NRF2/antioxidant response element (ARE) mechanism. Interestingly, activated NF-κB can bind directly on the Nrf2 promoter, regulating the transcription of the *NRF2* gene and, in turn, stimulating antioxidant response [18] (Figure 1). Intracellular TRX1 has been shown to be localized mainly in the cytoplasm; however, several factors such as reactive oxygen species (ROS), ionizing radiations, ultraviolet light, among other stimuli, have shown to induce the translocation of TRX1 to the nucleus, despite the lack of a nuclear localization signal [7]. The nuclear migration of TRX1 may, thus, be associated with the signaling molecules that connect the cytoplasmic and the nuclear events [7]. In this regard, we demonstrated that TRX1 levels, and its subcellular localization (i.e., cytosol and nucleus), were dependent on proteasome activity during the cell cycle, stress conditions, or the inhibition of the 20S proteasome [10]. 

Regarding the TRX2 isoform, it has been shown that its down-regulation impairs mitochondrial function, contributing to oxidative stress in mice [19]. It is noteworthy that mitochondrial TRX2, together with thioredoxin reductase 2 (TRXRD2), peroxiredoxin 3 (PRDX3) and peroxiredoxin 5 (PRDX5) [1,2], contribute to the elimination of H_2_O_2_, thus maintaining redox control of the mitochondrial matrix environment (Figure 1). Moreover, TRX2 is involved in controlling the intrinsic mitochondrial apoptotic pathway [20].

GLRXs are thiol-disulfide oxidoreductases and are part of the thioredoxin superfamily of proteins. GLRXs are glutathione (GSH)-dependent enzymes involved in the maintenance of cellular redox homeostasis by catalyzing reductions of disulfides or GSH-mixed disulfides (S-glutathionylation) in a coupled system which involves GSH, NADPH, and glutathione reductase (GR) [21]. Furthermore, GLRX can also act as Fe-S cluster transferases [22]. GLRX proteins react via monothiol or dithiol mechanisms using one or two cysteines in their Cys–Pro–Tyr–Cys active site [23,24,25,26]. Thus, they contribute to controlling the levels of internal disulfide bridges in proteins (by means of the di-thiol catalyzed mechanism) and reducing S-glutathionylation (using the mono-thiol mechanism) under conditions of oxidative stress [21,27] (Figure 1). Thanks to these mechanisms, GLRXs avoid the formation of irreversible Cys oxidations in functional proteins, and buffer reversible protein S-glutathionylation [28], which, in turn, protects cells against oxidative stress and controls redox signaling transduction [28]. GLRXs are almost ubiquitously present in a growing number of isoforms in most species [3]. In humans, GLRXs have been identified as two dithiol isoforms (i.e., GLRX1 and GLRX2) [29] and one mono- thiol GLRX (GLRX5 isoform) [30]. Cytosolic GLRX1 supports ribonucleotide reductase (RNR) with electrons to catalyze the formation of deoxyribonucleotides from ribonucleotides, and is involved in general disulfide-dithiol exchanges in proteins [31] and dehydroascorbate reduction [32] to regenerate a pool of reduced ascorbate and detoxify ROS. 

The mammalian GLRX2, which contains an iron-sulfur (Fe-S) cofactor [33], is present as two isoforms derived from alternative splicing forms: GLRX2a is targeted to mitochondria, whereas GLRX2b is localized in the nucleus [34,35]. The [2Fe-2S]^2+^ cluster in GLRX2 is coordinated by the Cys of the active sites of two monomers plus two additional non-covalently bound GSH molecules [29,30]. Interestingly, GSH used as a Fe-S ligand is continuously exchanged with free GSH present in the cytosol and mitochondria [36,37], suggesting a novel role of GSH in FeS cluster trafficking in which GLRX2 may participate. In fact, in a mammalian dopaminergic cell line model, depletion of GSH resulted in a defect of holo-GLRXs, which, in turn, produced a decrease in iron incorporation into mitochondrial complex I and aconitase [38]. Therefore, this (2Fe-2S-bridged dimers)-GSH complex is considered a redox sensor that could regulate oxidoreductase activity depending on the redox state of the cytosolic free glutathione pool [36]. In mitochondria, GLRX2 plays a central role in the response to oxidative stress and redox signaling by using S-glutathionylation as a regulatory mechanism [39]. In these subcellular organelles, GLRX2a exhibited higher affinity for glutathionylated substrates and electron donors compared with GLRX1, indicating a relevant role in regulating mitochondrial redox control by deglutathionylating target proteins such as the mitochondrial complex I [39,40]. It has also been shown that mitochondrial PRDX3 is reduced by GLRX2 [41] and TRX2, thereby contributing to mitochondrial detoxification of H_2_O_2_ (Figure 1). Furthermore, human GLRX2a seems to prevent cardiolipin oxidation and cytochrome *c* release from the mitochondria, thus protecting cells from apoptosis [42]. In the nucleus, the GLRX2b isoform demonstrated to perform several functions such as cellular differentiation [43], regulation of transcription factors [44,45,46] and support of the reduction potential of RNR during DNA synthesis [31]. 

Finally, human monothiol GLRX5, with high homology to yeast Glrx5p, contains a conserved active site with a Cys-Gly-Phe-Ser sequence and a N-terminal translocation signal to direct it to the mitochondrial matrix, where it is involved in the biogenesis of Fe-S clusters [47], thus linking GLRX5 to heme biosynthesis [30]. 

In summary, the maintenance of redox homeostasis in cells requires the restoration of the redox status of antioxidant proteins such as cytosolic TRX1, which are maintained in a reduced form by the GSH system thanks to the action of GLRXs [48,49] or by the action of thioredoxin reductase 1 (TRXRD1), although it has been demonstrated that TRXRD1 is not absolutely required to maintain reduced TRX1 levels in cells [50].

## 2. Thioredoxins and Glutaredoxins in Friedreich’s Ataxia

Friedreich’s ataxia (FRDA, MIM 229300) is an autosomal recessive neurodegenerative disease and it is the most prevalent hereditary ataxia in the Caucasian population, with a prevalence of around 2–4 in 100,000 individuals [51]. This rare childhood-onset disease is characterized by progressive spinocerebellar neurodegeneration, peripheral sensory neuropathy, vestibular and cerebellar pathology, and pyramidal disabilities in the last stages. All these disease-related alterations cause symptoms of gait and limb ataxia, lower limb areflexia and dysarthria in these patients [52]. Other non-neurological features of FRDA are scoliosis, diabetes and cardiac complications [53,54,55], which are the main cause of death in these patients, mostly in early adulthood. Most FRDA patients are homozygous for the GAA·TTC triplet repeat expansion in the *FXN* gene localized in chromosome 9q21.11 producing decreased protein levels of the protein product frataxin (FXN) [56,57]. Regarding the molecular characteristics of FRDA, there are well known alterations consisting of mitochondrial respiratory chain dysfunction [58], accumulation of mitochondrial iron [59], decreased mitochondrial DNA levels and adenosine triphosphate (ATP) generation, increased oxidative stress and unbalanced antioxidant response [60], as well as alterations in calcium homeostasis [61] and lipid metabolism [62,63]. 

Enzymatic antioxidant systems include superoxide dismutase Copper-Zinc superoxide dismutase (CuZnSOD) and Manganese superoxide dismutase (MnSOD), catalase, glutathione peroxidases, peroxiredoxins, and the TRX and GLRX systems, among others. Superoxide dismutase and catalase have previously been described as being altered in FRDA [64,65] and we have also found a deficiency in the expression of cytosolic CuZnSOD and mitochondrial MnSOD [60], which is in agreement with previous studies demonstrating that the up-regulation of MnSOD fails to occur in FRDA fibroblasts when they are exposed to iron [64,65]. However, despite the critical importance of the thioredoxin superfamily for cellular metabolism described above, there is little information on the specific role of TRX and GLRX systems in FRDA, and, therefore, we consider it to be of special relevance to elucidate their function in the molecular physiopathology of the disease.

The principal function of frataxin is still unknown; however, the involvement of the FXN protein in iron-sulphur clusters (Fe-S clusters), heme group biosynthesis [66], and mitochondriogenesis [67] has been reported, although only the role of the FXN protein in Fe-S cluster biogenesis seems to be more convincing and extensively proved [68]. The generation of iron-sulphur clusters and their insertion in apoproteins is a complex process that involves many players located in mitochondria and cytosol and divided into three sequential steps. In the first step, the [2Fe-2S] cluster is assembled on the scaffold protein iron-sulfur cluster assembly protein (ISCU2) from inorganic iron and sulfur. During this step, it has been proposed that frataxin interacts with ISCU2 and five other additional ISC proteins that form the ISC assembly complex. Furthermore, in the first studies, it was suggested that the FXN protein donated iron to the cluster [2Fe-2S] [69,70]. Posterior experiments proposed frataxin as an allosteric regulator for sulfur transfer to the Fe-S cluster [71,72,73], although currently the main mechanism accepted is a preloading of ISCU2 with iron [74]. Dysregulation of FXN protein function in ISC assembly can produce several alterations in cells, including those produced by deficits in Fe-S cluster-containing mitochondrial enzymes, such as aconitase and succinate dehydrogenase. In fact, low levels of aconitase, an enzyme from the tricarboxylic acid cycle (TCA cycle), and mitochondrial respiratory complexes I, II, and III have been determined in frataxin-deficient animal and cellular models [61]. These alterations lead to metabolic changes that decrease ATP generation in the mitochondrion. In addition, studies on FRDA patients [58] and cellular [60] and animal models [58,75,76] showed mitochondrial dysfunction and lower ATP levels. Another important enzyme that contains a Fe-S cluster is ferrochelatase (FECH), which catalyzes the last step of heme group biosynthesis, where the iron atom is incorporated into protoporphyrin IX. Previously, an iron atom should be provided to FECH through a process that is not yet known. The involvement of FXN in this process has been reported by in vitro analysis and one study in yeast that showed an FXN protein interaction with ferrochelatase [77]. Nevertheless, the role of frataxin in heme group biosynthesis is controversial and the last suggested model of mitochondrial heme metabolism did not include this protein [78]. These results are in agreement with a recent analysis in erythroid progenitor cells from FRDA patients, in which heme synthesis was not altered during erythroid differentiation [79].

Iron homeostasis and iron-sulfur cluster biosynthesis are closely related processes. Indeed, impaired FeS-dependent activities and an activation of IRP1 (iron regulatory protein 1) have been described in the liver of frataxin-deficient mice, increasing iron import and availability by promoting gene expression of the iron-response element (IRE) containing promoter genes [80]. Iron accumulation in the spleen, liver, and heart has been described in FRDA patients [81] and animal models [82,83], thus suggesting altered iron metabolism in FRDA. However, controversial studies about iron accumulation in neural tissues can be found in the literature [84,85,86,87]. The implication of iron accumulation in the physiopathology of FRDA is not yet clarified, and further analyses are needed to address this issue, especially regarding neural degeneration in FRDA. However, the newly described process of ferroptosis has provided a possible mechanism for neuronal death, since it explains many of the pathological characteristics of neuronal degeneration in FRDA. Ferroptosis is a regulated cell death that is distinct from other cell death processes, such as apoptosis, classical autophagy, and necrosis. Ferroptosis is characterized by an overwhelming, iron-dependent accumulation of lethal lipid hydroperoxides [88]. It has been suggested that the initiation of ferroptosis might be directly triggered by an increase in free iron levels, for example by a dysregulation of ferritinophagy, a selective autophagy of ferritin [89]. Iron increase or accumulation induces the Fenton reaction which promotes the production of ROS, and together with the lipoxygenase activity of 15-LOX (ALOX15), oxidizes polyunsaturated fatty acids phospholipids (PUFA-PLs) which activate the ferroptosis pathway [90,91]. In addition, inhibition of glutathione peroxidase enzyme 4 (GPX4) [92,93] or GSH unavailability or defects in its restoration [88,94] produce lipid hydroperoxide accumulation that triggers ferroptosis. Importantly, the implication of TRX1 and TRXRD in ferroptosis has also been described [95,96]. Increased ROS, lower reduced GSH concentrations and enhanced sensitivity to oxidants compared with control neurons have also been observed in these FRDA cell models [97,98]. Part of ROS production occurs in the mitochondria as a consequence of the malfunction of respiratory complex I [99]. Importantly, through the mitochondrial one-carbon metabolism, NADPH production is severely compromised when the function of Complex I is affected [100], as occurs in blood cells from FRDA patients [101,102]. The compromised levels of NADPH may affect cellular thiol-based redox regulation because the classical thioredoxin system is composed of TRX, TRXRD and NADPH, which are required as electron donors for TRXRD [103] and glutathione reductase to replenish GSH levels, which are used by glutaredoxins [4] and GPX4 to reduce lipoperoxides [104,105].

In relation to this, FRDA neurons have shown higher lipoperoxide levels, increased ROS, lower reduced GSH concentration, and enhanced sensitivity to oxidants compared with control neurons [97,98]. Neurons from a YG8R mouse model also showed a mitochondrial energy imbalance, as a consequence of an inhibition of mitochondrial Complex I and increased lipid peroxidation, which contribute to cell death [106]. Furthermore, patients with FRDA present a disturbance of GSH homeostasis [107,108,109], lipoperoxidation and thiol oxidation [110,111]. Together with iron accumulation, all these results suggest the occurrence of ferroptosis in FRDA.

### 2.1. TRXs and GLRXs Are Downregulated in FRDA Models

In yeast models of FRDA (Δyfh1 cells), the anaerobiosis to aerobiosis transition is an inducer of oxidative stress as a result of transcriptional repression of several genes which encode for antioxidant enzymes such as SODs, catalases, GLRXs, and TRXs [112,113]. TRXs and GLRXs dysfunction may produce several deleterious effects on the physiopathology of FRDA as a consequence of their role not only in antioxidant defense but also in additional molecular processes such as metabolism, cell function and survival. In fact, besides the function of TRXs and GLRXs in modulating oxidative stress by regulating transcription factors such as NRF2 [114,115], these thioredoxin family proteins can also control iron metabolism, particularly Fe-S clusters [116]. Gene expression analysis and analysis of protein levels of dorsal root ganglia in an FRDA mouse model (YG8R mice) revealed significant differences in genes belonging to the thiol antioxidant system (including the *TrxR* and *Gpx* systems) [108]. The authors showed a significant decrease in the expression of Prdx3 and Grx1 proteins, as well as in the expression of the *Txn2* gene and the thioredoxin-interacting protein (*Txnip*) gene (the negative regulator of the biological function and expression of TRX by direct interaction). In addition, it has been shown that deficits in selenium metabolism may also alter the ability of TRXR to re-establish the reduced state of TRXs in FRDA [108,117]. These results are in accordance with the main pathological mechanism described in FRDA, oxidative stress. 

**Figure 2:**
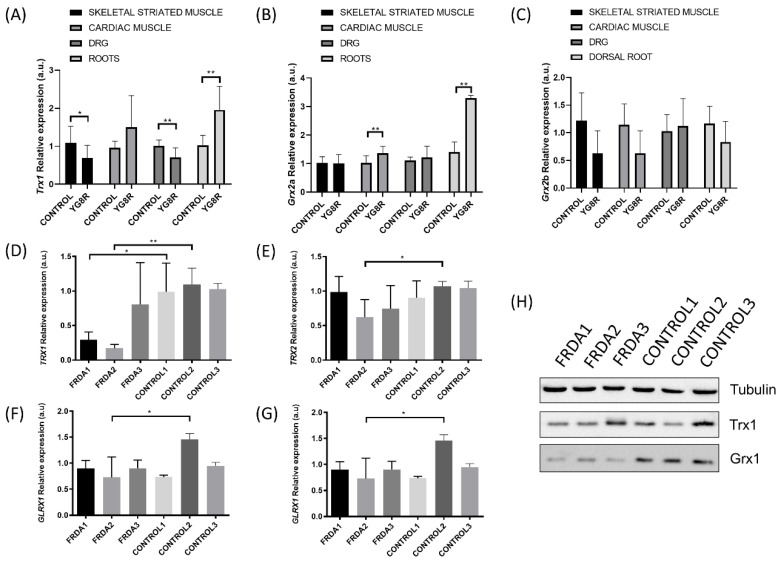
Changes in the levels of Trx and Glrxs expression in mice and human fibroblast Friedreich’s ataxia (FRDA) models. The relative mRNA expression of Trx1 (**A**), Glrx2 isoform a (**B**), and Glrx2 isoform b (**C**) was determined in skeletal striated muscle, cardiac muscle, dorsal root ganglia (DRG), and nerve roots of a C56B6LJ control mouse (*n* = 3) and YG8R frataxin-deficient mouse model (*n* = 3). The relative mRNA expression of TRX1 (**D**), TRX2 (**E**), GLRX1 (**F**) and GLRX2 (**G**) was analyzed in fibroblasts from FRDA patients (*n* = 3) and healthy subjects (*n* = 3). The relative mRNA expression levels were calculated using Glyceraldehyde-3-Phosphate Dehydrogenase (GAPDH) as an endogenous control. (**H**) Western blot analysis of protein levels for TRX1 and GLRX1 in fibroblasts from FRDA patients and healthy subjects. Statistical significance refers to the values for the CONTROL samples for each tissue (* *p* < 0.05, ** *p* < 0.01). Methodology is described in Supplementary Materials.

The results obtained by Shan et al. suggest that deficits in frataxin contribute to disruption in antioxidant protection in the YG8R hemizygous mouse model dorsal root ganglia (DRG) [108]. We extended the study of gene expression of the TRX and GLRX systems to other tissues to determine the oxidative damage in YG8R. We found that *Trx1* gene expression was downregulated in skeletal striated muscle (Figure 2A); however, gene expression levels of the mitochondrial isoform *Glrx2a* increased in cardiac muscle (Figure 2B). Kenchappa and Ravindranath explored how GLRX1 activity is important in maintaining mitochondrial function and demonstrated that downregulation of Glrx1, but not of GSH, leads to mitochondrial complex I dysfunction [118]. While Glrx1 seems to play an important role in preserving mitochondrial integrity, thereby preventing complex I activity loss, GLRX2 could restore complex I activity in mitochondria by catalyzing the deglutathionylation of Cys residues [119]. Interestingly, inhibition of hu- man Grx2 by means of siRNA technology decreased mitochondrial complex I and aconitase activities in a mammalian dopaminergic cell line [38]. Perhaps the overexpression of *Glrx2a* that we observed in cardiac muscle could be a response to the need for increasing deficient mitochondrial respiration in a tissue with high bioenergetic metabolism.

On the other hand, the effects of FXN deficiency on TRX and GLRX activities in neural tissues are controversial. DRG from YG8R mice showed a decrease in *Trx1* mRNA levels but an increase in nerve roots (equivalent to axons) (Figure 2A). Regarding *Glrx2a* mRNA levels, we did not observe differences in DRG, but the expression in nerve roots also increased. Thus, this differential expression could be associated with the location inside of the same cell in structures as specialized as the axon. Interestingly, GLRX1 can catalyze the deglutathionylation of actin, which is crucial for its polymerization in cellular dynamics. Pastore et al. demonstrated how actin S-glutathionylation caused an impairment of microfilament organization in FRDA fibroblasts [120]. This mechanism gains special relevance in neurons, since the S-glutathionylation of actin seems to be a key process involved in their protection, as accumulation of disarranged actin filaments in turn affects neuronal development and trafficking [121]. Similarly, analysis of the GLRXs and TRX systems in FRDA1 and FRDA2 fibroblast cell lines from FRDA patients showed decreased expression of TRX1 and GLRX1 when compared to their respective control fibroblasts (Figure 2), which may compromise the correct activity of key enzymes in these cells obtained from FRDA patients. This significant reduction in antioxidant genes and proteins may be related to the downregulation of NRF2 expression, which has previously been described in the DRG of YG8R [114] and in fibroblasts from FRDA patients [122]. NRF2 expression is primarily responsible for the NRF2-mediated protection of neurons from oxidative stress by activating the transcription of antioxidant enzyme codifying genes [123], such as antioxidant enzymes (i.e., SODs, catalase, GPXs), GSH synthesis enzymes (i.e., GCL expression), and activating the ARE, a cis-acting regulatory element found in the promoter regions of genes encoding for phase II detoxification enzymes such as NADPH quinone oxidoreductase (NQO), HO-1, glutathione S-transferase (GST) [124,125,126] and the TRX and TRXRD [114,127,128].

### 2.2. TRX1 Nuclear Translocation Is Altered in FRDA Fibroblasts

It is widely accepted that the NRF2 signaling pathway is altered in FRDA [108,129,130,131]. In fact, NRF2 is regulated by its inhibitor Kelch-like ECH-associated protein 1 (KEAP1), which has been described to be importantly overexpressed in FRDA models [129]. NRF2 modulates redox imbalance in the cell through the regulation of antioxidant response [132]. The interplay between NRF2 and thioredoxin superfamily enzymes is complex, because both systems interact with each other. That means that the TRX system is activated by NRF2, but, in turn, the TRX system acts as a modulator of the KEAP1-NRF2 response pathway [50]. NRF2 has been shown to be downregulated in human biopsies such as in blood cells of 26 children with FRDA [122], and defects in its nuclear translocation have also been found in FRDA fibroblasts [130] and in motor neuron-like cells [131]. 

The mechanism underlying KEAP1-NRF2 regulation to maintain the redox status of the cell involves the participation of at least two redox-sensitive Cys residues within nuclear localization signal (NLS) and nuclear export signal (NES) sequences in NRF2, which become affected as a consequence of oxidative stress. In fact, the oxidation of these Cys alters NRF2 translocation into the nucleus to activate ARE elements. In this scenario, Cys oxidation may be reversed by nuclear GSH and TRX systems, as has been described for TRX1 [133]. In particular, the subcellular compartmentation of NRF2 is dependent on the concentration of cytosolic free GSH (which promotes NRF2 dissociation from KEAP1) and nuclear TRX1 (which promotes NRF2-DNA binding) thereby contributing to the control of transcriptional regulation mediated by NRF2/ARE [133]. In this regard, cells containing less cytosolic or nuclear GSH [134], low TRX1 levels in the nucleus may develop altered NRF2/ARE signaling. Interestingly, it has been stablished that differentiated cells that contain less GSH would be prone to activating the NRF2 pathway even with milder oxidative stress [133], and it has also been proposed that cellular antioxidant activity mediated by NRF2/ARE signaling may be different in proliferating cells as compared to differentiated cells. In the first cells, which contain higher GSH concentrations and high GSH/GSSG ratios [135], NRF2 may not be as rapidly activated as in differentiated cells [133]. Finally, it is noteworthy that the data obtained from FRDA cellular models highlight an abnormal distribution of actin fibers causing defective binding of the NRF2–KEAP1 complex and the failure of NRF2 nuclear translocation [130], which may be synergistically affected by two additional mechanisms: (1) the downregulation of TRX1, which may reduce the ability of TRX1 to bind to ARE domains; and (2) the downregulation of GRX1, which is necessary to produce the deglutathionylation of actin which is disarranged in FRDA, thereby altering microfilament organization in FRDA cells [120], which, in turn, is necessary for nuclear translocation of NRF2.

**Figure 3:**
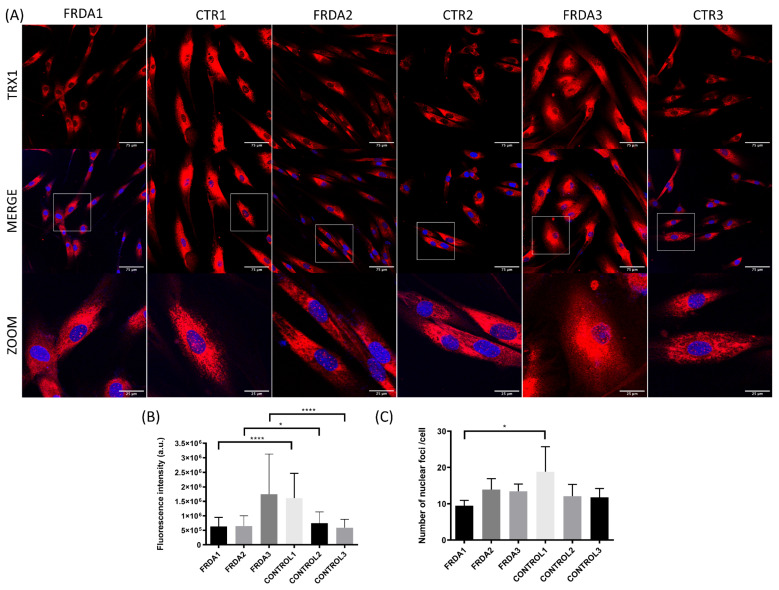
Subcellular localization of TRX1 in fibroblasts from FRDA patients (FRDA1, FRDA2, and Figure 3. *n* = 3) and from matched healthy subjects (CTR1, CTR2, and CTR3; *n* = 3). (**A**) Representative images of fibroblasts stained with anti-TRX1 antibody from each patient and healthy subject. Confocal images show the maximal intensity projection. The images were used to determine the subcellular localization of TRX1 (red). The nuclei were stained with Hoechst (blue), and zooms show the colocalization nuclear region. (**B**) Quantification of relative fluorescence intensity of TRX1 from confocal images in each cell line of fibroblasts from FRDA patients and controls. Fluorescence intensity was measured for each cell in at least three different images of each cell line using ImageJ software. The Mann–Whitney test was used to compare each patient with its paired control to obtain statistical significance. (**C**) Quantification of the number of nuclear foci of TRX1 per cell. The total number of nuclear foci was measured in cells with the entire nuclei and was normalized by the number of total cells. At least three different images of each cell line were measured using ImageJ software. The Mann–Whitney test was used to compare each patient with a control to obtain statistical significance. The statistical analyses were performed using Graphpad prism 8.0 software. Statistical significance refers to the values for the matched healthy subject for each FRDA patient (* *p* < 0.05, **** *p* < 0.0001). Methodology is described in Supplementary Materials.

We have explored the levels of total and nuclear TRX1 using anti-TRX1 antibody and confocal microscopy. We observed a decrease in fluorescence intensity for TRX1 in FRDA1 and FRDA2 fibroblast cell lines compared to control fibroblasts (Figure 3A,B). Interestingly, one of the fibroblast cell lines (FRDA1) showed a decreased number of nuclear foci of TRX1 compared to control fibroblasts (Figure 3A,C). This result is consistent with our previously published data regarding reduced antioxidant response (i.e., Cat, Gpx1 and MnSOD) in this FRDA fibroblast cell line obtained from a 30-year-old male with a GAA triplet expansion (370/470) and mild peripheral neuropathy and cardiomyopathy [60].

### 2.3. Thioredoxin and Glutaredoxin Systems in Iron and Iron-Sulfur Cluster Metabolism

Intra-cellular and extra-cellular iron levels need to be tightly controlled, and, therefore, organisms have developed effective mechanisms for iron (either in a free or complexed form) regulation. The homeostasis of iron is of utmost importance to human health, and its dysregulation can lead to various disorders. Recent research has suggested that iron accumulation-induced dysfunction of the iron regulatory proteins and the iron-responsive element (IRP-IRE) signaling pathway contribute to protein aggregation and neuron loss in neurodegenerative diseases such as Alzheimer and Parkinson diseases [136].

In complex organisms, iron homeostasis is maintained by iron regulatory proteins (IRP1 and IRP2) and the IRE signaling pathway [137,138], and it is noteworthy that thioredoxin family proteins are important mediators in iron metabolism since these proteins regulate the expression of IRPs. IRPs bind to RNA stem-loops containing an IRE within the untranslated region (UTR) of mRNA transcripts to regulate the translation of mRNAs, the products of which are involved in iron metabolism, including ferritin heavy chain (*FTH1*) and ferritin light chain (*FTL*), divalent metal transporter 1 (*DMT1*), transferrin receptor protein 1 (*TFRC*), transferrin (*TFR*) aconitase (*ACO2*), ferroportin-1 (*SLC40A1*), among others. However, iron can bind to IRPs, leading to the dissociation of IRPs from the IRE and thereby alter the translation of target transcripts.

In FRDA, progressive cardiomyopathy (the main cause of death in these patients) is, in part, produced by iron-catalyzed mitochondrial damage followed by muscle fiber necrosis and chronic reactive myocarditis. In this regard, accumulation of IRPs such as cytosolic ferritin, mitochondrial ferritin, and ferroportin have been found in heart tissue of FRDA patients [139]. Moreover, neuronal atrophy and a peculiar proliferation of synaptic terminals in the dentate nucleus termed “grumose degeneration” have been reported in the brain of FRDA patients. Koeppen et al. studied IRP levels in the dentate nucleus and found that these regions of grumose degeneration were strongly reactive for ferroportin, as also occurred in Purkinje cell bodies, their dendrites and axons [84], suggesting that grumose degeneration is the final morphological manifestation of mitochondrial iron metabolism dysregulation. Importantly, grumose degeneration is closely related to the loss of FXN from synaptic terminals [140].

With these precedents, it is obvious that IRP cellular-mediated processes are of special relevance in FRDA, and, therefore, any other processes controlling IRP function may produce direct consequences in iron metabolism. In this regard, it was recently demonstrated that TRX1, particularly its [2Fe-2S] cluster, is required for the reduction of cysteinyl residues in apo-IRP1, a prerequisite for the binding of IRP1 to IREs [141], and contribute to the translation of proteins involved in iron metabolism. This result is of particular relevance since it points out the role of TRX1 as a key protein which links redox metabolism and the function of iron-related proteins.

A subclass of GLRX containing the active site Cys-Ser/Gly-Tyr-Cys (C-S/G-Y-C) can also act as Fe-S cluster transferases. This active site (C-S/G-Y-C) bridges two GLRX2 molecules to form a dimeric holo-GLRX2 complex with the thiols of two non-covalently bound GSH [33,37]. However, the GLRX2 complex can be activated after receiving redox signals (such as the detection of low levels of GSH) to reduce disulfide-bridges with GSH to free thiol groups in the Cys, which, in turn, activate the oxidoreductase activity of GLRX2 [33,36]. The human monothiol GLRX3 and GLRX5 have a relevant role in iron homeostasis and Fe-S cluster biosynthesis [116,142]. 

Interestingly, an impaired Fe-S cluster assembly that resulted in heme biosynthesis was observed in a human sideroblastic-like microcytic anemia in a patient with reduced GRX5 levels, thereby reinforcing the idea that thioredoxin superfamily proteins are relevant in Fe-S biosynthesis and homeostasis [30].

All these processes in which TRX and GRX participate in Fe-S cluster biogenesis, assembly and function seem to be very relevant in FRDA. Their function could be essential in a disease in which frataxin is deficient, since this protein has been proven to be involved in Fe-S cluster biogenesis [143] Therefore, correct Fe-S cluster biogenesis in mitochondria is intimately linked to cellular iron homeostasis, and failure in the assembly of mitochondrial Fe-S proteins upon frataxin deficiency results in increased iron import, availability and accumulation in mitochondria after the activation of IRP1 [68]. 

The role of thioredoxin family proteins remains an unexplored field in FRDA, but further efforts should be made in order to clarify the specific implications of TRXs and GRXs malfunction and, in particular, their involvement in Fe-S clusters or their contribution with frataxin in Fe-S cluster biogenesis in mitochondria and how they participate to the appropriate function of IRPs and iron metabolism, all of which are crucial in the physiopathology of FRDA [144]. 

### 2.4. Thioredoxin and Glutaredoxin Systems Regulating Ferroptosis

**Figure 4:**
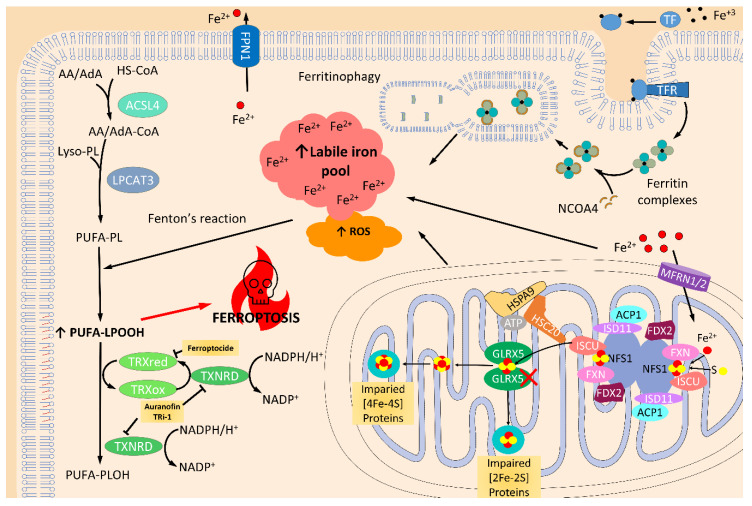
Ferroptosis activation by the inhibition of the thioredoxin and glutaredoxin systems. Ferroptosis is programed cell death produced by overwhelmed, and iron-dependent, lipid peroxidation accumulation. As a consequence, iron trafficking, storing, and related metabolism, such as iron-sulfur (Fe/S) cluster (ISC) biosynthesis, are key players in ferroptosis. Glutaredoxin 5 (GLRX5) is essential for mitochondrial ISC protein biogenesis. Indeed, inhibition of this protein truncates the transference of ISC to mitochondrial [2Fe-2S] and [4Fe-4S] proteins and, as a result, produces impaired mitochondrial function, which in turn increases the reactive oxygen species (ROS) levels [157]. Furthermore, iron that is not used in ISC protein production is accumulated and increases the levels of the labile iron pool. Together, increased iron levels and elevated ROS levels, promote Fenton’s reaction which raises the amount of oxidized polyunsaturated fatty acid phospholipids (PUFA-PLOOH), and triggers ferroptosis by a mechanism not well known. Detoxification of these oxidized lipids is done by glutathione peroxidase 4 (GPX4) [154], as well as thioredoxin (TRX) and thioredoxin reductase (TXNRD) [158,159]. In fact, it has recently been demonstrated that inhibition of these proteins can produce increased levels of ROS, lipid peroxidation, and ferroptosis activity [95,146]. Transferrin receptor (TFR); transferrin (TF)-iron import; ferroportin (FPN1); nuclear receptor coactivator 4 (NCOA4); acyl-CoA synthetase long-chain family member 4 (ACSL4); arachidonic acid (AA); adrenoic acid (AdA); lysophosphatidylcholine Acyltransferase 3 (LPCAT3); cysteine desulfurase (NFS1); LYR motif-containing protein 4 (ISD11); mitochondrial acyl carrier protein (ACP1), ferredoxin (FDX1/2), and frataxin (FXN); mitochondrial iron-sulfur cluster assembly enzyme (ISCU); iron-sulfur cluster co-chaperone protein HscB (HSC20); mitochondrial stress-70 protein(HSPA9); mitoferrin-1/2 (MFRN1/2). ↑ indicates increased levels.

TRXs and GLRXs have opened new avenues in the research of ferroptosis-mediated cellular death and its relevance in neurons [145,146,147]. A recent study by Llabani et al. found a new ferroptocide compound that rapidly and robustly induces ferroptosis by inhibiting thioredoxin activity [95]. In addition, a study performed in C57BL/6J mice treated with auranofin (a pan-TRXRD inhibitor) and TRi-1 (a specific TRXRD1 inhibitor) reported that the inhibition of TRXRD could trigger lipid peroxidation and ferroptosis [148]. Another recent study on breast cancer cells reported that auranofin increased lysine oxidase (LOX)-mediated cytotoxicity in an ROS-dependent mechanism [149] by activating caspase-independent cell death mediated by necroptosis and ferroptosis (Figure 4). Surprisingly, in a recent pre-print study, it is claimed that the TXNRD1 inhibition in pancreatic cancer cells confers protection against ferroptosis by increasing GPX4 protein levels [150], which is an inhibitor of ferroptosis [88,151,152,153,154] and is mainly involved in the detoxification of lipoperoxides thanks to its anti-lipoperoxidant action [153,154,155,156]. While it seems that TRX proteins are relevant mediators in ferroptosis, little knowledge is currently available on the role of GRXs in ferroptosis. Only one recent study described that genetic silencing of GLRX5 increases lipid peroxidation, ROS production, free iron accumulation and ferroptosis in head and neck cancer (HNC) cells [157] (Figure 4). Altogether, these recent studies point to the relevance of TRXs and GRXs in modulating ferroptosis. Nonetheless, the specific mechanism of ferroptosis regulation is still poorly understood, and further experiments are needed to clarify the role of every component of these families. 

In the previous section, we showed that a reduction in antioxidant genes and proteins in FRDA may be related to the downregulation of NRF2 expression, which has previously been described in DRG of YG8R [114] and in fibroblasts from FRDA patients [122], and this is of special relevance because the NRF2-ARE signaling pathway is required for TRX and TRXRD expression [114,127,128]. Thus, the association between the downregulation of TRX and TRXRD in FRDA and the recent findings of the role of TRXRD in ferroptosis strongly suggest the importance of this mechanism in FRDA.

Ferroptosis has been shown to occur in a number of central nervous system (CNS) disorders including Parkinson’s [160] and Alzheimer’s disease [161]. Interestingly increased peroxidation of phospholipids, which is consistent with the occurrence of ferroptotic damage in inflammatory demyelinating disorders, has also been described [152]. In this regard, it has been proposed that the hypomyelination or demyelination in neurons of FRDA may be a consequence of defects in Schwann cells [162,163], although ferroptosis may also directly participate in this process.

## 3. Activation of the Thioredoxin Family by NRF2 Activators as Therapeutic Options in FRDA

As shown above, it is well established that oxidative stress plays a key role in the pathophysiology of FRDA both by means of unbalanced antioxidant enzymatic and non-enzymatic responses. Therefore, for many years, antioxidants have been evaluated as potential therapeutic agents for FRDA, and some authors have recently reviewed potential therapies based on antioxidant strategies [164]. Among the different therapies evaluated, overexpression of NRF2 seems to be a promising approach to promote antioxidant response in FRDA, and hence, several trials using omaveloxolone or resveratrol in order to overexpress NRF2 and stimulate ARE activation have been proposed [164]. 

Targeted therapies to stimulate the expression of TRX have a wide array of beneficial effects in neurodegenerative disorders and other hyperinflammatory diseases in which the expression or function of these proteins are altered. Preclinical and clinical studies using recombinant TRX (rhTRX) are currently underway, although there are also natural substances (including active principles from plants) which can induce the expression of thioredoxin family proteins [13]. Yodoi et al. reviewed the most promising strategies to deliver TRX as a therapeutic agent, including (i) topical application, (ii) oral delivery, and (iii) TRX-overexpression using exogenous stimuli [13]. Topical applications may have little relevance for neurological diseases, but oral delivery and TRX-overexpression can be considered feasible therapeutic strategies in neurodegenerative disorders such as FRDA. Nevertheless, it is more plausible to use an indirect strategy to induce TRX superfamily overexpression. Thus, as described in the previous section, it would be possible to activate TRX by upregulating the stability, expression, and activation of NRF2. In this regard, a recent review by La Rosa, Bertini and Piemonte described the pharmacological interventions aimed at restoring the NRF2 signaling network in FRDA [165]. Among the several molecules described to stimulate NRF2 overexpression, resveratrol was found to increase both NRF2 stability and mRNA overexpression of NRF2 [166,167] and, in turn, TRX1 [168,169]. 

Resveratrol has been proposed as a potential antioxidant treatment in FRDA and as an inducer of frataxin expression. In FRDA mouse models and cells from FRDA patients (i.e., fibroblasts and lymphoblasts), resveratrol treatment demonstrated an ability to increase the transcription of a stably transfected frataxin-green fluorescent protein [170]. However, these results were not reproduced in peripheral blood mononuclear cells obtained from FRDA patients [171] nor in induced pluripotent stem cell (hPSC)-derived neurons from patients with FRDA [172]. Moreover, an open-label trial using low-dose (1 g daily) and high-dose resveratrol (5 g daily) in FRDA patients, despite suggesting clinical benefits for high-dose resveratrol, did not demonstrate an increase in frataxin levels in FRDA patients [171]. Interestingly, in a model of ischemia-reperfusion of liver, trans-resveratrol demonstrated the ability to maintain TRX redox activity by diminishing TXNIP protein expression and, more importantly, the ability to inhibit the secretion of the TRX1 protein [173]. The same results were observed in an in vivo model of old mice with or without 3-month resveratrol treatment [174]. These results suggest that the expression of TRX can ameliorate the symptoms of FRDA probably by improving some of the mechanisms we have described in the previous section, despite not increasing the expression of frataxin levels.

Compared with resveratrol, sulforaphane (SFN) more potently activates NRF2 to induce the expression of the antioxidant system [175]. SFN is an isothiocyanate derived from glucoraphanin, which is mainly found in cruciferous vegetables such as broccoli, Brussels sprouts, cabbages and cauliflower. Its potential to increase the expression and activity of NRF2 and TRX1 has been demonstrated by ARE transcription activation in murine retina [176]. Interestingly, Jazwa et al. showed that intra-peritoneal injection of SFN can cross the blood–brain barrier in the MPTP mouse model of Parkinson’s disease, being detected in the brain 15 min after injection [177]. Besides its potential to increase the expression of NRF2 and TRX1 in some cellular models such as retinal cells [176] and human hepatoma cells [178], SFN also demonstrated its ability to increase the expression of TRXRD, and together with selenium helps to increase the activity of TRXRD [178]. The reactivation of TRXRD may serve to re-establish the pool of reduced TRX and maintain antioxidant homeostasis in cells, which, in turn, may contribute to the release of NRF2 from KEAP1 [50], thereby activating the transcriptional function of NRF2. It is noteworthy that Chiang et al. also found that SFN can increase the expression of both NRF2 and its inhibitor KEAP1 in a SK-N-MC neuroblastoma cell line, which could be explained as a feedback mechanism to prevent NRF2 overexpression and its downstream antioxidant defense genes [179].

When SFN treatment was evaluated in frataxin-silenced motor neuron-like cells [180], neural stem cells isolated from the KIKO FRDA mouse model [181] and also in FRDA fibroblasts [182], this antioxidant was able to revert the cellular phenotypic defects, providing neuroprotection in the neuronal models. Unfortunately, despite these findings, SFN has not yet been evaluated in clinical trials for FRDA.

Omaveloxolone is another inductor of NRF2 expression able to reverse the FRDA phenotype in different pre-clinical models. Omaveloxolone protects the cells against oxidative stress, avoids lipid peroxidation, decreases the mitochondrial ROS generation, and increments reduced glutathione levels [183]. Recently, results from a clinical trial with this drug have been published pointing out that omaveloxolone significantly improves neurological function and is generally safe and well tolerated [184].

Finally, melatonin has been defined as a principal regulator of Nrf2 signaling and improves oxidative stress state (reviewed in [185]). Moreover, melatonin has been described as an endoplasmic reticulum stress mediator, promoting TRX1 activity by inhibiting the TXINP/NLRP3 pathway [186]. Despite the fact that melatonin has been described as a possible treatment in other neurodegenerative diseases [187], in FRDA, only one case report has been described. In this case, the authors of the study administrate 5 and 10 mg of melatonin to an FRDA patient to treat REM (rapid eye movement phase of sleep) Sleep Behavior Disorder; however, they did not find any benefit after melatonin treatment [188].

The activation of thioredoxin superfamily proteins through NRF2 activators (such as omaveloxolone, resveratrol, sulforaphane and melatonin) can represent promising therapeutic options in FRDA, and, as such, they have been or are already being subject of pre-clinical and clinical trials (Table 1). The reason is that the activation of NRF2 and in turn TRXs and GRXs may contribute to decreased oxidative stress in FRDA cells, to improve the metabolism of iron-sulfur clusters required for appropriate mitochondrial metabolism, to decrease iron-catalyzed mitochondrial damage and also to inhibit ferroptosis, all of them related with the molecular pathogenesis in FRDA. We consider that further efforts exploring therapeutic candidates overexpressing NRF2 and thioredoxin family proteins may increase the therapeutic strategies for this neuromuscular disease.

## 4. Conclusions

The data provided in this review illustrate that unbalanced antioxidant enzymatic and non-enzymatic responses in which thioredoxin family proteins have a key role are main actors in the molecular physiopathology of FRDA. TRX and GRX have relevant roles in the mechanisms occurring in FRDA, including decreased antioxidant response, alterations in mitochondrial metabolism and Fe-S cluster synthesis, defects in NRF2 signaling, alterations in IRP-IRE signaling and ferroptosis. In this regard, therapeutic approaches focused on increasing the expression of thioredoxin family proteins may be useful in FRDA. Moreover, antioxidants such as resveratrol and SFN have important implications in key mechanisms underlying FRDA as activators of TRX family proteins [164,189,190]. The widespread use of antioxidants as a therapeutic approach in FRDA has led patients and caregivers to believe that antioxidant drugs are useful [191]. However, no clear clinical evidence has been demonstrated so far. The current tendency is directed to the use of treatment strategies designed to avoid oxidative stress, increase cellular frataxin, improve mitochondrial function as well as modulate frataxin-controlled metabolic pathways, avoiding endoplasmic reticulum stress and defects in calcium homeostasis so as enable the recovery of iron homeostasis, among others [164,189]. 

Therefore, comprehension of these mechanisms may help to design more adequate clinical trials in FRDA involving the administration of single drugs or combined therapies to simultaneously revert different downstream effects produced by frataxin deficiency, and in which proteins from the TRX family undoubtedly would play a prominent role.

## Figures and Tables

**Table 1:** Pre-clinical and clinical antioxidant therapies in FRDA.

Compound	Pre-Clinical Studies in FRDA			Clinical Trials in FRDA		
	Model	Doses/Treatment	Ref.	Nº Subjects	Doses/Treatment	Ref.
Resveratrol	YG8R mouse	200 mg/kg daily for 3 days. Subcutaneous injection.	[170]	27 FRDA patients: 13 low dosis and 14 high dosis	0.5 g or 2.5 g twice daily for 12 weeks. Capsules.	[171]
	Human fibroblast MSCiPSC-derived neurons	25 µM to 125 µM once 25 µM to 125 µM once 10 µM to 50 µM once	[172]	40 patients (estimated)	2 g daily for 24 weeks. Capsules.	ClinicalTrials.gov Id: NCT03933163
Sulforaphane (SFN)	Mouse NSC34 motor neurons Human fibroblasts	5 µM for 24 h 10 µM for 24 h	[180]			
	Neural stem cells KIKO mouse	5 µM for 2, 6, and 24 h	[181]			
	Human fibroblast	10 µM for 2, 6, and 24 h	[182]			
Omaveloxolone	Cerebellar Granule Neurons KIKO and YG8R mice Human Fibroblast	50 nM for 24 h 50 nM for 24 h	[183]	103 patients	150 mg daily for 48 weeks. Capsules.	[184]
Melatonin				Case report: 1 FRDA patient	5 mg and 10 mg	[188]

## Supplementary Materials

The following are available online at https://www.mdpi.com/2076-3921/9/12/1257/s1, Supplementary material and methods.
