# Supplementary material for: Thioredoxin and Glutaredoxin Systems as Potential Targets for the Development of New Treatments in Friedreich’s Ataxia
Source: Antioxidants (Basel). 2020 Dec 10;9(12):1257. doi: 10.3390/antiox9121257 (PMC7763308; doi:10.3390/antiox9121257)
Supplement: Supplementary file 1 [file antioxidants-09-01257-s001.pdf]

Supplementary material.

1. Methods

a. Cell culture

FRDA fibroblasts (GM04078, GM03816 and GM03665) and control fibroblasts (GM08402 and GM01652) were obtained from Coriell Cell Repository (Camden, NJ), and Control 3 was kindly donated by Dr. Marcela Del Río (Supplementary Table 1). Characteristics and clinical aspects of patients have described previously [1]. The cells were cultured in Eagle's minimum essential medium with Earle's salts and non-essential amino acids (MEM, Gibco, Invitrogen) supplemented with 10% fetal bovine serum inactivated and 1% penicillin-streptomycin (Sigma-Aldrich, St. Louis, MO) in 5% CO_2 in air at 37 ºC at density of 20.000 cells/cm^2. The subcultivation method used was trypsin-EDTA. Studies were performed at cell confluence.

b. Expression studies

RNA isolation

Total RNA was isolated from cells using the PARIS™ Protein and RNA Isolation System (Ambion; Catalog # 1921; Austin, TX) according to the manufacturer's instructions.

cDNA synthesis

For reverse transcription reactions (RT), 1 μg of the purified RNA was reverse transcribed using random hexamers with the High-Capacity cDNA Archive kit (Applied Biosystems, P/N: 4322171; Foster City, CA) according to the manufacturer's instructions. RT conditions comprised an initial incubation step at 25ºC for 10 min. to allow random hexamers annealing, followed by cDNA synthesis at 37ºC for 120 min, and a final inactivation step for 5 min. at 95ºC.

Measurement of mRNA Levels

The mRNA levels were determined by quantitative real-time PCR analysis using an ABI Prism 7900 HT Fast Real-Time PCR System (Applied Biosystems, Foster City, CA). Gene-specific primer pairs and probes for *TXN* (Hs01555214_g1), *TXN2* (Hs00429399_g1), *GLRX1* (Hs00829752_g1), *GLRX2* (Hs00375015_m1), *Trx1* (Mm00726847_s1), *Glrx2* (Mm00469836_m1) and *Glrx2a* (Mm01291253_m1) (Assay-on-demand, Applied Biosystems), were used together with 1x TaqMan® Universal PCR Master Mix (Applied Biosystems, P/N 4304437; Foster City, CA) and 2 μl of reverse transcribed sample RNA in 20 μL reaction volumes. PCR conditions were 10 min. at 95ºC for enzyme activation, followed by 40 two-step cycles (15 sec at 95ºC; 1 min at 60ºC). The levels of glyceraldehyde-3-phosphate dehydrogenase (*GAPDH*: Hs02786624_g1; *Gapdh*: Mm99999915_g1) expression were measured in all samples to normalize gene expression for sample-to-sample differences in RNA input, RNA quality and reverse transcription efficiency. Each sample was analyzed in triplicate, and the expression was calculated according to the $2^{-\Delta\Delta Ct}$ method [2].

c. Cell lysates and Western blot analysis

Cell lysates

Approximately 3 ×·10^6 cells were lysed using lysis buffer (Hepes, pH 7.4, 20 mM, tritonX-100 1%, NaCl 100 mM, NaF 50 mM, β-glycerophosphate 10 mM, activated sodium orthovanadate 1 mM, PMSF 1mM, protein proteases inhibitor cocktail 2 μL/mL in ice about 15 min and then the suspension was centrifugated at 13000 g for 10 min at 4 ºC and the supernatants were collected and stored at -80 ºC until their use.

To obtain cytosolic cell lysate cellular lysis buffer (5mM HEPES pH 8.0, 85 mM KCl, 0.5% NP40) was added on the flask, after 15 min incubation on ice, the cells were scraped, harvested in 15 mL tubes and spun for 5 min at 3000 rpm at 4ºC. The supernant, consisting in cytosolic cell lysate, was collected in 1,5 mL tubes and the pellet formed by nuclei was break down with 100 L of nuclear lysis buffer (50 mM TrisHCl pH 8.1, 10 mM EDTA, 1% SDS). Both lysates were stored at -20ºC until their use.

d. Western blot analysis

Protein content was determined by a modified Lowry method. Aliquots of cell lysates (40-50 μg) were added to sample buffer with 10% *β*-mercaptoethanol and then were immediately boiled for 5 min and separated by electrophoresis in sodium dodecyl sulfate 12% polyacrylamide gels (SDS-PAGE), 100V during two hours. After electrophoresis, the proteins were electroblotted (Bio-Rad) onto nitrocellulose membrane. Membranes were blocked with 0.05g/mL non-fat milk or BSA 0.05g/mL in TBS-0.2% Tween 20 (TBST) according to the antibody, washed three times at room temperature, and incubated with primary antibodies against thiorredoxin 1, (1:1000, Santa Cruz BioTech., TX, USA), glutharredoxin 1 (1:1000, Abcam, Abcam, MA, USA.), and tubulin (1:1000, Santa Cruz BioTech, TX, USA) antibody as the loading control, in TBST with 0.01 g/mL non-fat milk during 2 h at room temperature. Thereafter, the blots were washed again with TBST and further incubated for 1 h with a secondary mouse or rabbit) conjugated with horseradish peroxidase-linked. After washing with TBST as above, blots were developed by using the ECL™ Western Blotting Detection Reagents as specified by the manufacturer (Amersham GE Healthcare). Chimioluminiscent signals were assessed using a Fujifilm scanning densitometer (Fujifilm LAS-1000 plus). The relative optical density (ROD) was quantified by Image J (National Institutes of Health, Bethesda, MD, USA).

e. Immunofluorescence analysis

FRDA and control cells were plated in 2cm^2 LAB-TEK II chambered cover glass (Nunc, Thermo Fischer Scientific, Waltham, 138 MA, USA). Cells were fixed with 4% paraformaldehyde in PBS containing 10% fetal calf serum for 20 min at room temperature, followed by permeabilization for 10 min using 0.2% (w/v) Triton X-100 in PBS. After incubation with primary antibodies (TRX1) overnight at 4ºC) The slides then were incubated for 1 h with a secondary antibody as follows: fluorescein isothiocyanate-conjugated sheep antimouse IgG (Amersham Pharmacia Biotech) for TRX, or tetramethylrhodamine isothiocyanate-conjugated goat anti-rabbit IgG (Sigma). Hoechst 2 μg/mL (Sigma-Aldrich, St. Louis, MO, USA) was used to localize nuclei. Slides with stained cells were examined with a confocal microscope Leica TCS-SP2 confocal laser scanning unit equipped with argon and helium-neon laser beams and attached to a Leica DM1RB inverted microscope (*Leica* Microsystems, Mannheim, 135 Germany). The maximum projection was obtained from Z-series. The sum of pixels was selected as a parameter for fluorescence intensity (RawIntDen). To determinate the subcellular localization of TRX1 the number of nuclear foci per cell were quantified.

f. Animals

The experiments were performed using the YG8R FRDA mouse model purchased from The Jackson Laboratory Repository (Stock no. 008398). The YG8R mouse consists in an *Fxn* knockout, homozygous for a deletion of the exon 4 of the *Fxn* gene (embrionary lethality), rescued by the presence in hemizygosis of transgene including the human *FXN* gene with a pathological number of GAA repeats (main mutation in FRDA patients). Animals were maintained and selected from a colony of YG8RxYG8R as previously described [3]). All handling and protocols were carried out following the practices established and approved by the Bioethics subcommittee of *Consejo Superior de Investigaciones Científicas* (CSIC; Supplementary Table 1).

g. Statistical analysis

Each cell model was independently compared with respect to their CONTROL cell line applying a t- test. The results represent the mean±SD of three independent experiments. The P-values under

0.05 were considered significant. The error bars inside graphs represent the standard deviation of the replicate samples. A statistical data analysis was performed using the GraphPad Software v6.0 (GraphPad Software, San Diego, CA, USA).

References

1. Garcia-Gimenez, J.L.; Gimeno, A.; Gonzalez-Cabo, P.; Dasi, F.; Bolinches-Amoros, A.; Molla, B.; Palau, F.; Pallardo, F.V. Differential expression of PGC-1alpha and metabolic sensors suggest age-dependent induction of mitochondrial biogenesis in Friedreich ataxia fibroblasts. *PLoS One* **2011**, *6*, e20666, doi:10.1371/journal.pone.0020666.
2. Livak, K.J.; Schmittgen, T.D. Analysis of relative gene expression data using real-time quantitative PCR and the 2(-Delta Delta C(T)) Method. *Methods* **2001**, *25*, 402-408, doi:10.1006/meth.2001.1262.
3. Molla, B.; Riveiro, F.; Bolinches-Amoros, A.; Munoz-Lasso, D.C.; Palau, F.; Gonzalez-Cabo, P. Two different pathogenic mechanisms, dying-back axonal neuropathy and pancreatic senescence, are present in the YG8R mouse model of Friedreich's ataxia. *Dis Model Mech* **2016**, *9*, 647-657, doi:10.1242/dmm.024273.

Supplementary Table

Supplementary Table 1. Cell and mouse models used in this work.

Name of sample	Repository ID	*Specie*
**FRDA1**	**GM04078**	***Homo sapiens***
**FRDA2**	**GM03816**	***Homo sapiens***
**FRDA3**	**GM03665**	***Homo sapiens***
**CONTROL1**	**GM08402**	***Homo sapiens***
**CONTROL2**	**N/A**	***Homo sapiens***
**CONTROL3**	**GM01652**	***Homo sapiens***
**C57BL6J**	**N/A**	***Mus musculus***
**YG8R**	**008398**	***Mus musculus***
